# Gas Crosstalk between PFPE–PEG–PFPE Triblock Copolymer Surfactant-Based Microdroplets and Monitoring Bacterial Gas Metabolism with Droplet-Based Microfluidics

**DOI:** 10.3390/bios10110172

**Published:** 2020-11-11

**Authors:** Sunghyun Ki, Dong-Ku Kang

**Affiliations:** 1 Department of Chemistry, Natural Science, Incheon National University, Incheon 22012, Korea; shki@inu.ac.kr; 2 Research Institute of Basic Sciences, Incheon National University, Incheon 22012, Korea

**Keywords:** droplet microfluidics, PFPE–PEG–PFPE surfactant, crosstalk between droplets

## Abstract

The PFPE–PEG–PFPE (Perfluoropolyether-polyethylene glycol-perfluoropolyether) surfactant has been used in droplet-based microfluidics and is known to provide high droplet stability and biocompatibility. Since this surfactant ensures the stability of droplets, droplet-based microfluidic systems have been widely used to encapsulate and analyze various biological components at the single-molecule scale, including viruses, bacteria, nucleic acids and proteins. In this study, we experimentally confirmed that gas crosstalk occurred between droplets formed by fluorinated oil and the PFPE–PEG–PFPE surfactant. *E. coli* K-12 bacterial cells were encapsulated with Luria–Bertani broth within droplets for the cultivation, and gas crosstalk was identified with neighboring droplets that contain phenol red. Since bacteria produce ammonia gas during its metabolism, penetration of ammonia gas initiates a color change of phenol red-containing droplets. Ammonia gas exchange was also confirmed by reacting ammonium chloride and sodium hydroxide within droplets that encapsulated. Herein, we demonstrate the gas crosstalk issue between droplets when it is formed using the PFPE–PEG–PFPE surfactant and also confirm that the density of droplet barrier has effects on gas crosstalk. Our results also suggest that droplet-based microfluidics can be used for the monitoring of living bacteria by the determination of bacterial metabolites during cultivation.

## 1. Introduction

Droplet-based microfluidics (DMF) has been continuously developed in biomedical engineering and analytical chemistry to monitor various chemical and biological analytes such as viruses, bacteria, nucleic acids and proteins [1,2,3,4,5]. It provides various advantages such as high-throughput, short reaction time, higher efficiency and cost-effectiveness for analysis of interesting biological targets [6,7,8,9,10]. In addition, DMF cannot only generate monodisperse droplets but also control the flow rate to generate droplets of various sizes. That is why it has been widely used in various research fields such as single fundamental biology, diagnostics and high-throughput drug screening [11,12,13,14,15].

For single-molecule analysis using DMF, droplets must be kept stable for various reactions, including thermocycling and long incubation. Oils and surfactants used for the encapsulation must be highly biocompatible and also can provide stability in droplets to avoid breakage and crosstalk between droplets. Recently, various surfactants and oils have been explored in DMF for sample partitioning and also for various applications [16]. One of the most commercially successful application may be the droplet digital PCR (ddPCR) which requires thermocycling and also need to be perfectly separated during reaction [4,7,11,13,17]. Among various surfactants, PFPE-based surfactants have been most commonly used in DMF because of their excellent long-term stability and biocompatibility with biological systems [18,19,20,21,22]. Weitz and his colleagues demonstrated the biocompatibility and stability of PFPE–PEG–PFPE surfactants in various applications [18,23]. However, there are no studies on gas crosstalk between droplets.

Therefore, in this study, gas crosstalk was first validated between droplets generated with HFE-7500 and the PFPE–PEG–PFPE surfactant. To summarize the overall experimental process, First, phenol red was encapsulated within droplets as a pH indicator to analyze the crosstalk between droplets. *E. coli* K-12 was also encapsulated within droplets in a single-cell manner to validate the crosstalk with phenol red droplets after mixing and incubation. These results suggest that pH indicators or bacterial metabolites can cross the droplet barrier. Then bacterial metabolite was identified as transferring component between droplets, and it was characterized as an ammonia gas with Nessler’s reagent [24,25,26,27]. Finally, ammonia gas was artificially produced through a chemical reaction between ammonium chloride and sodium hydroxide within droplets to experimentally validate that the ammonia gas can actually transport into neighboring droplets. As a result, artificially produced ammonia gas can penetrate the droplet barrier, and the phenol red droplet turned red due to pH change. We also confirmed that surfactant density is a major reason that affects the gas permeability between droplets by validation with droplets that were generated with various concentrations of surfactant. Basically, a higher concentration of surfactant reduced gas penetration between droplets and these results suggest that surface density is the major barrier to gas permeability. These results suggest that live bacteria and bacterial growth can be identified in a single-cell manner by monitoring the penetration of ammonia gas from bacterial cell-containing droplets to indicator droplets.

## 2. Materials and Methods

### 2.1. Materials

HFE-7100, oxalyl chloride, anhydrous dichloromethane, Jeffamine ED-900, phenol red, trichloro(1H,1H,2H,2H-perfluorooctyl)silane, sodium hydroxide, ammonium chloride were purchased from Sigma-Aldrich (St. Louis, MO, USA). A Krytox 157-FSH was purchased from Chermours (Wilmington, DE, USA). HFE-7500 was purchased from 3 M (Saint Paul, MN, USA). SU-8-negative photoresist and SU-8 developer were purchased from Kayaku advanced materials (Westborough, MA, USA) for the fabrication of master mold. A Sylgard 184 silicone elastomer kit was purchased from Dow Corning (Midland, MI, USA) for the fabrication of microfluidic devices as polydimethylsiloxane (PDMS) material. Luria–Bertani (LB), Luer-Lok syringe and 25G syringe needle were purchased from BD (Franklin Lakes, NJ, USA). Microfluidic devices were punched with a 1 mm biopsy punch (Kay Industries Co., Gifu, Japan) to provide inlets and outlets. A Nessler’s reagent (potassium tetraiodomercurate (II)) was purchased from Duksan science (Seoul, Korea). NanoDrop™ 2000c spectrophotometer (Thermo Fisher Scientific, Waltham, MA, USA) was used to determine the concentration of bacterial cells. Plasma cleaner PDC-002 (Harrick Plasma, Ithaca, NY, USA) was used to bond the PDMS layer with the glass slide by O_2_ plasma treatment. To analyze droplets under microscope, a countess cell counting chamber slide (chamber slide) was purchased from Invitrogen (Waltham, CA, USA). Aqueous and oil were introduced with syringe pumps (PHD ultra, Harvard Apparatus, Holliston, MA, USA). For droplet generation, droplets were imaged and recorded under a DMi-8 fluorescence microscope (Leica, Wetzlar, Germany) with a DFC-7000T CCD camera (Leica, Wetzlar, Germany) of Leica Microsystems (Wetzlar, Germany). Microscopic images were analyzed using LAS X software (Version 3.4.2) and image J software (Version 1.52t) from Leica Microsystems.

### 2.2. Process of Synthesis of PFPE–PEG–PFPE Surfactant

**Figure 1:**
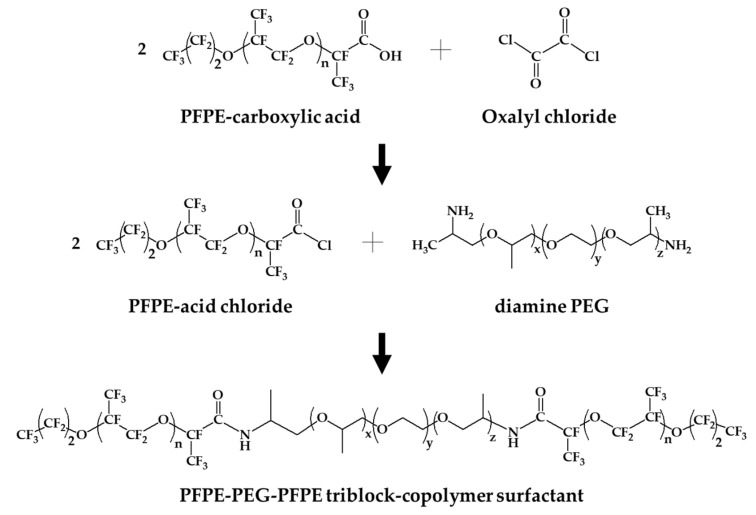
Schematic diagram of the synthesis process for PFPE–PEG–PFPE triblock copolymer surfactant.

**Figure 2:**
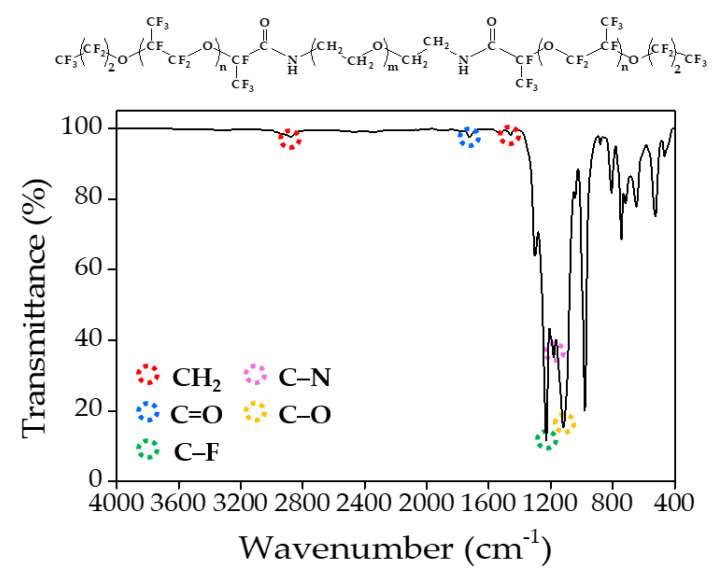
ATR-FTIR spectrum of synthesized surfactant for structural analysis.

**Figure 3:**
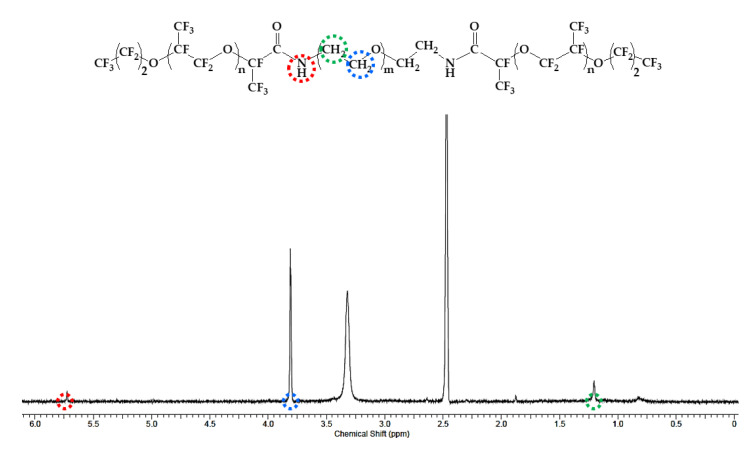
^1^H-NMR spectrum of synthesized PFPE–PEG–PFPE surfactant for structural analysis.

**Figure 4:**
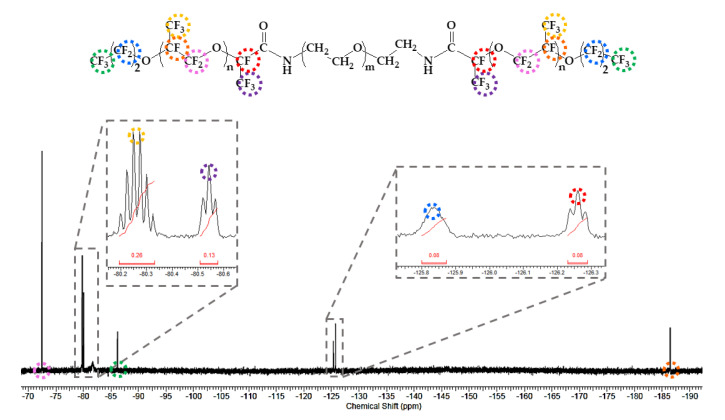
^19^F-NMR spectrum of synthesized PFPE–PEG–PFPE surfactant for structural analysis.

The synthetic process of PFPE–PEG–PFPE surfactant was mainly performed by composing in two steps (Figure 1), which are distinguished in (step 1) a substitution from carboxylic acid to acid chloride and (step 2) an amide bonding process. First, Krytox 157-FSH (2.85 mmol, ~7000 g/mol) was dissolved with HFE-7100 and mixed with oxalyl chloride (28.3 mmol, 126.93 g/mol). Then, oxalyl chloride was added 10 times, comparing the volume of Krytox 157-FSH. The mixture was stirred and incubated overnight at 85 °C under a nitrogen atmosphere, and then it could be seen that the product became slightly yellow. Remaining residues of unreacted oxalyl chloride and HFE-7100 were removed under a rotary evaporator. Then, the remaining product was dissolved in HFE-7100 for the reaction with a diamine PEG (1.5 mmol, Jeffamine ED-900) was dissolved in anhydrous dichloromethane for the central hydrophilic block of the surfactant. The mixture was stirred and incubated for 48 h at 65 °C under a nitrogen atmosphere. After 48 h incubation, the mixture was transferred in a 50 mL conical tube for centrifugation at 8000 rpm (or higher) for 10 min to remove residues of white particles. Then, the final product was dried in a vacuum desiccator for 24 h [18,28]. For the structure determination, the final product was analyzed with ATR-FTIR spectroscopy (Spectrum two, Perkin-Elmer, Waltham, MA, USA) (Figure 2), ^1^H-NMR (Figure 3) and ^19^F-NMR spectroscopy (400-MR, Agilent Technologies, Santa Clara, CA, USA) (Figure 4) [29].

### 2.3. Fabrication of Microfluidic Chip

Silicon wafer-based molds of microfluidic devices were fabricated by conventional photolithography methods [30,31]. Film photomasks were designed for microfluidic devices with AutoCAD software (version 2018), and four-inch silicon wafers were used as a substrate for the microfluidic devices. First, silicon wafers were cleaned using a piranha solution (3:1 concentrated sulfuric acid to 30% hydrogen peroxide) and then 1% hydrogen fluoride to remove debris and oxide layer. Silicon wafers were then patterned with negative photoresist SU-8 50 at 2000 rpm (30 s) using a spin coater. After pre-baking at 65 °C for 6 min, photoresist-coated silicon wafers were soft-baked at 95 °C for 20 min. The photoresist was cured under a wavelength of 400 nm for 23 s at a power of 11 mW/cm^2^. Then polymerization was performed at 65 °C for 1 min as a post-exposure baking (PEB) and then baked at 95 °C for 5 min. Unpolymerized photoresist was removed using a SU-8 developer and washed with 100% hexane, 100% methanol and 100% isopropanol. Dried wafers were dried using a Trichloro (1H,1H,2H,2H-perfluorooctyl)silane in a desiccator overnight. In this experiment, microfluidic chips were fabricated with PDMS [32,33,34]. Briefly, PDMS base and curing agent were mixed in a ratio of 10:1 (*w*/*w*%), and the mixture was then degassed under desiccator prior to pouring onto silicon wafer-based master mold. It was then polymerized on a 65 °C hot plate overnight. After baking, the PDMS layer was peeled off from the master mold and punched with a 1 mm biopsy punch to provide inlets and outlets. PDMS layer and glass slides were initially treated with an oxygen plasma before there were bonded, and bonded devices were incubated on the hot plate at a temperature of 85 °C for 15 min.

### 2.4. Cell Culture and Droplet Generation

As a model system to prove bacterial metabolism within microdroplets, *Escherichia coli* (*E. coli*) K-12 was used in this study. A single colony was obtained from freshly grown on the agar plate containing LB, and it was then inoculated in 5 mL LB broth at 37 °C overnight. Ten (10) μL of bacterial culture was further inoculated in 5 mL fresh LB broth with shaking at 37 °C for 4 h, and the concentration of bacterial cells was determined by NanoDrop™ 2000c spectrophotometer prior to encapsulation.

**Figure 5:**
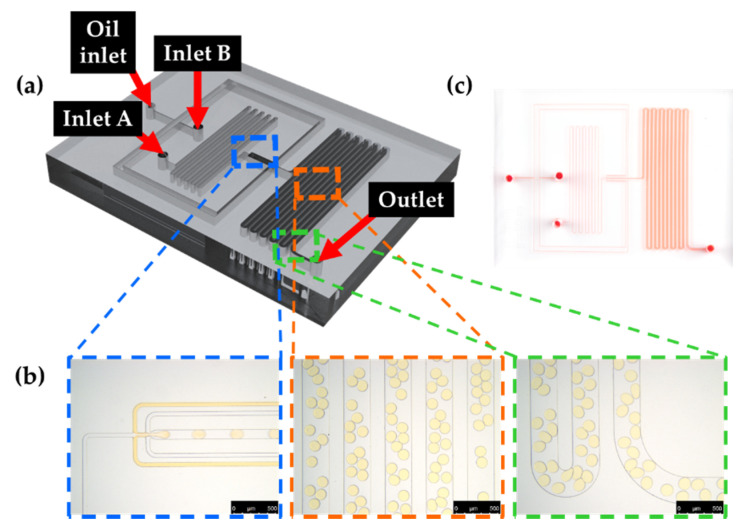
Droplet generation using the microfluidic device. (**a**) Schematic diagram of the microfluidic device for droplet generation. (**b**) Microscopic images of flow-focusing structure (blue box) for droplet generation, incubation channels (orange box) and outlet (green–box) (scale bar = 500 μm). (**c**) A photographic image of the fabricated PDMS microfluidic device. The microfluidic channel was visualized with red food coloring.

A microfluidic device contains an outlet, an oil-phase inlet and two aqueous-phase inlets. To identify ammonia gas crosstalk from bacterial cells, *E. coli* K-12 was encapsulated with HFE-7500 containing 2% (*w*/*w*) surfactant. To mimic ammonia gas production within droplets, 2 M sodium hydroxide and 2 M ammonium chloride were also encapsulated within droplets (ammonia gas generating droplets) using HFE-7500 with 2% surfactant. *E. coli* K-12-containing droplets or ammonia gas generating droplets were then mixed with 1 mg/mL phenol red droplets to identify gas permeability between droplets with chamber slide. More briefly, synthesized surfactant was dissolved in HFE-7500 at a concentration of 2% and introduced through oil inlet. In aqueous phase, *E. coli* K-12 or sodium hydroxide/ammonium chloride was introduced through aqueous inlets and then encapsulated with HFE-7500 at the flow-focusing structure within microfluidic devices (Figure 5) [35]. Droplets were generated to be approximately 80 μm by the optimum flow rate of the oil and aqueous phase. Generated droplets were collected in 1.5 mL tubes for further analysis.

### 2.5. Detection of E. coli K-12 with Phenol Red, pH Indicator

**Figure 6:**
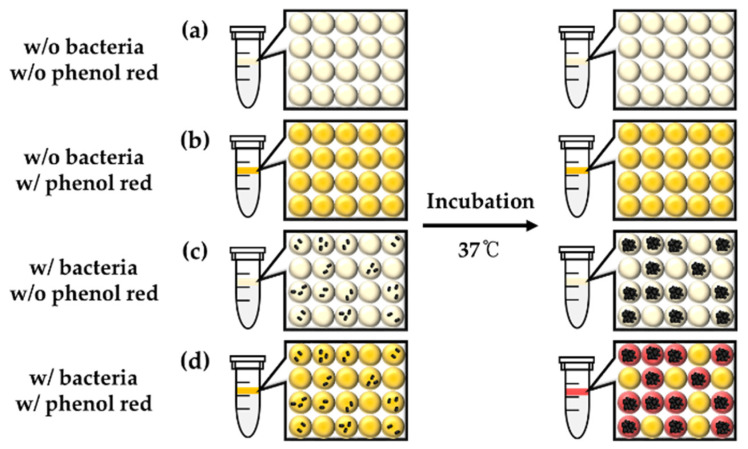
Schematic diagram of the experimental plan to determine bacterial growth by cultivation within droplets using s pH indicator. (**a**) Empty droplets, (**b**) phenol red droplets, (**c**) bacteria droplets, (**d**) phenol red and bacteria droplets for the incubation and detection by the color change of phenol red.

**Figure 7:**
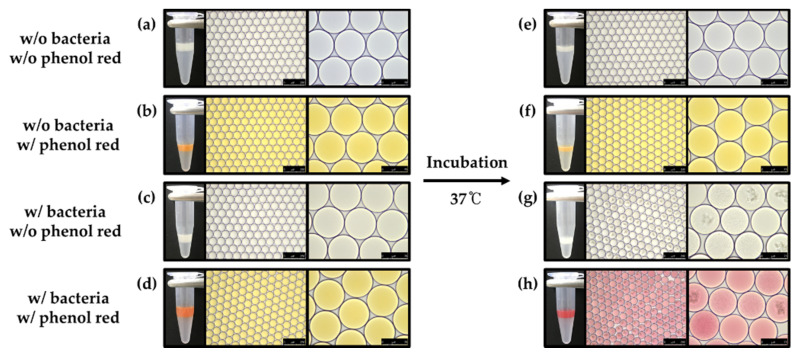
Identification of bacterial growth in droplets using pH indicator. (**a**,**e**) Empty droplet, (**b**,**f**) phenol red droplets, (**c**,**g**) bacteria droplet, (**d**,**h**) co-encapsulated droplets of both phenol red and bacteria (scale bar of left panels = 250 μm, scale bar of right panels = 75 μm).

*E. coli* K-12 was cultured in fresh LB broth at 37 °C overnight and introduced into the microfluidic devices to be encapsulated within 80 μm droplets in a single-cell manner with 1 mg/mL phenol red using HFE-7500 containing 2% surfactant. More briefly, LB broth was encapsulated into microdroplets with or without 1 mg/mL phenol red. LB broth containing *E. coli* K-12 was also encapsulated with or without 1 mg/mL phenol red to identify bacteria-induced color exchange within droplets (Figure 6). Generated droplets were collected with 1.5 mL tubes and droplets were incubated at 37 °C. After overnight cultivation, droplets were then imaged under a DMi-8 microscope to identify color exchange (Figure 7).

### 2.6. Identification of Crosstalk between Droplets

**Figure 8:**
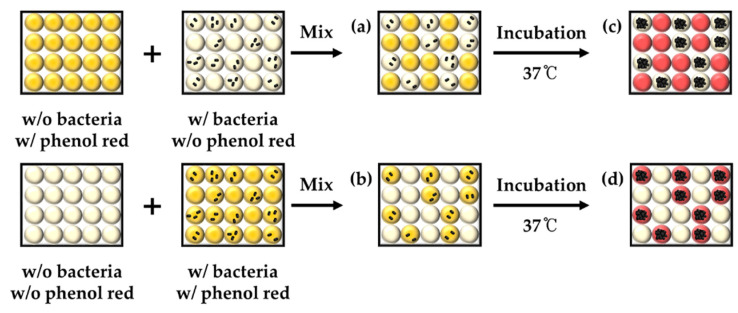
Schematic diagram of crosstalk between mixed droplets. Two types of droplets were mixed and incubated for identifying the gas crosstalk.

**Figure 9:**
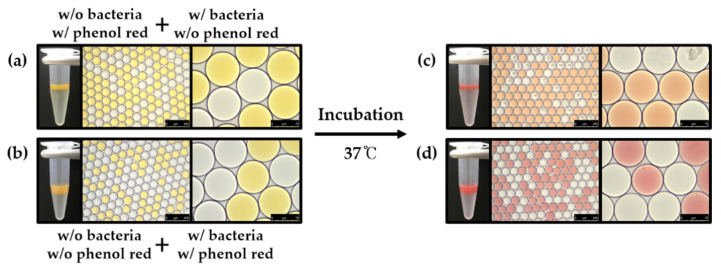
Identification of crosstalk components through droplet barrier. (**a**) Droplet mixture of phenol red-containing droplets with *E. coli* K-12-containing droplets. (**b**) Droplet mixture of empty droplets with co-encapsulated droplets (both phenol red and *E. coli* K-12). Droplet mixtures were imaged before (**a**,**b**) and after (**c**,**d**) overnight incubation. (scale bar of left panels = 250 μm, scale bar of right panels = 75 μm).

*E. coli* K-12 was freshly cultured in LB broth at 37 °C overnight and encapsulated within 80 μm droplets in a single-cell manner without phenol red. Phenol red was separately encapsulated in 80 μm droplets at a concentration of 1 mg/mL as an indicator of pH change to define crosstalk between droplets. Each droplet was mixed and incubated overnight prior to the identification of metabolite transfer through the droplet barrier (above panels, Figure 8 and Figure 9). To identify phenol red dye permeability between droplets, fresh LB broth was also encapsulated within droplets without *E. coli* K-12 and 1 mg/mL phenol red to be mixed with droplets that contain both *E. coli* K-12 and phenol red. Mixtures of droplets were incubated at 37 °C overnight. Droplets were then imaged under a DMi-8 microscope (below panels, Figure 8 and Figure 9).

### 2.7. Identification of Ammonia Gas Production in Bacterial Cells

**Figure 10:**
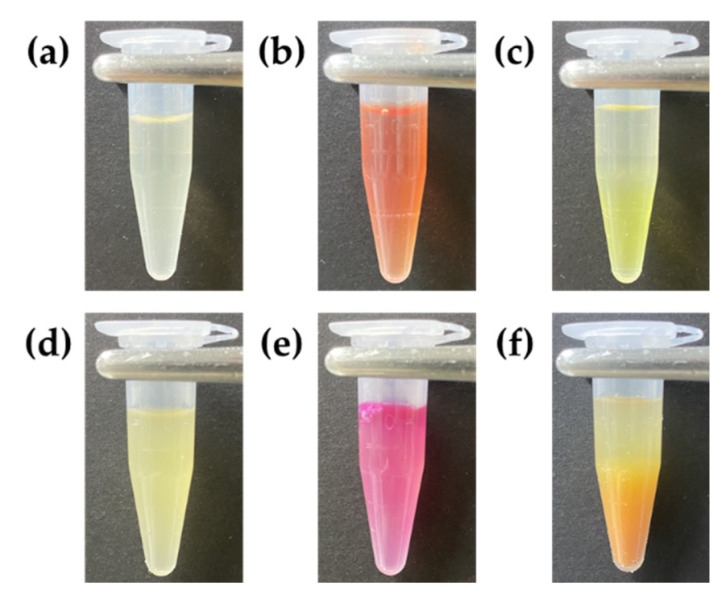
Identification of bacterial metabolite with phenol red and Nessler’s reagent. (**a**) Only LB broth, (**b**) LB broth with phenol red, (**c**) LB broth with Nessler’s reagent, (**d**) *E. coli* K-12 (**e**) *E. coli* K-12 with phenol red and (**f**) *E. coli* K-12 with Nessler’s reagent.

In order to confirm ammonia gas production during bacterial cell cultivation, ammonia gas was detected with Nessler’s reagent, which is a conventional indicator for ammonia gas, and it was compared with phenol red dye in bulk. *E. coli* K-12 was cultured in LB broth, and 1 mL *E. coli* K-12-containing LB broth was transferred into 1.5 mL tubes. One milliliter of fresh LB broth was also prepared as a negative control as a bacteria-free condition, and 100 μL 100% Nessler’s reagent or 100 μL 1 mg/mL phenol red added. The color change was analyzed immediately with a smartphone camera (iPhone 11 pro, Apple, Cupertino, CA, USA) (Figure 10).

### 2.8. Validation of Ammonia Gas Crosstalk between Droplets

**Figure 11:**
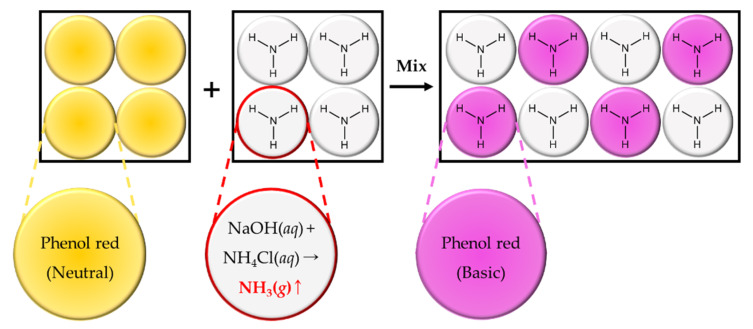
Schematic diagram of artificially produced ammonia gas within droplets and its crosstalk between droplets.

**Figure 12:**
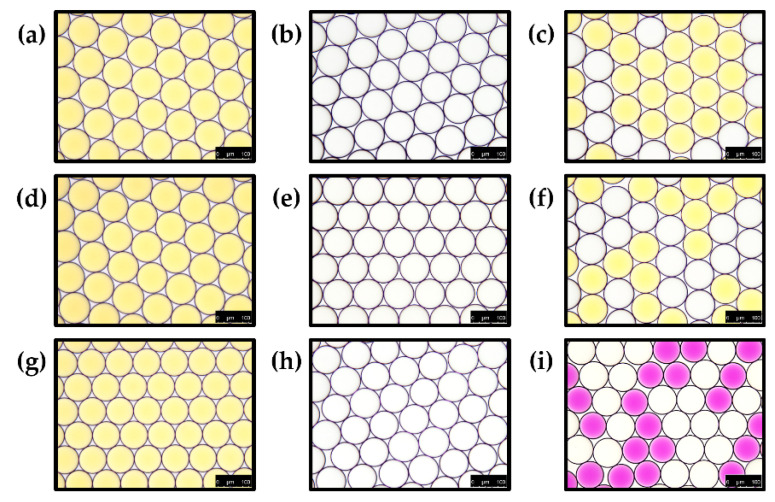
Identification of ammonia gas production within droplets and gas crosstalk between droplets. Phenol red droplets (**a**,**d**,**g**) were mixed sodium hydroxide-containing droplets (**b**), ammonium chloride-containing droplets (**e**) or co-encapsulated droplets with sodium hydroxide and ammonium chloride (**h**). Droplet mixtures (**c**,**f**,**i**) were then imaged under a DMi-8 microscope (scale bar = 100 μm).

As a model system of ammonia gas production by chemical reaction within droplets (ammonia gas generating droplets), 2 M sodium hydroxide and 2 M ammonium chloride were co-encapsulated within 80 μm droplets [36]. Phenol red was also solely encapsulated within LB broth at a concentration of 1 mg/mL as indicator droplets to determine the transfer of ammonia gas through the droplet barrier (Figure 11). Ammonia gas generating droplets (10 μL) and indicator droplets (10 μL) were transferred within the same tubes and gently mixed for 10 s prior to imaging under a DMi-8 microscope. A mixture of droplets was then imaged immediately without incubation. To identify gas production through the chemical reaction between sodium hydroxide and ammonium chloride, 2 M sodium hydroxide or 2 M ammonium chloride were separately encapsulated before mixing with indicator droplets (Figure 12). All procedures were performed at room temperature (23 °C) in this experiment.

### 2.9. Effect of Ammonia Gas Concentration on Gas Permeability

**Figure 13:**
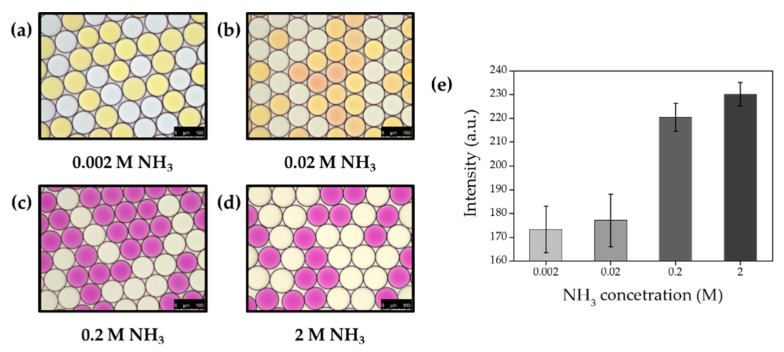
Permeability on ammonia gas concentration between droplets. Ammonia gas generating droplets were generated separately at ammonia gas concentration of 0.002 M (**a**), 0.02 M (**b**), 0.2 M (**c**) and 2 M (**d**) and mixed with indicator droplets, respectively. (**e**) Mean values of color exchange of indicator droplets for ammonia gas concentration (scale bar = 100 μm).

To identify the effect of gas concentration on permeability between droplets, sodium hydroxide and ammonium chloride were co-encapsulated at a different concentration from 0.002 M to 2 M. Droplets containing different concentrations of sodium hydroxide and ammonium chloride were separately collected within 1.5 mL tubes. Indicator droplets (10 μL) were also prepared with 1 mg/mL phenol red to be mixed with ammonia gas generating droplets (10 μL). Mixed droplets were immediately transferred into the chamber slide for imaging under a DMi-8 microscope, and the microscopic image was analyzed with the image J software (Figure 13). All procedures were performed at room temperature (23 °C) in this experiment.

### 2.10. Effect of Surfactant Concentration on Gas Permeability

**Figure 14:**
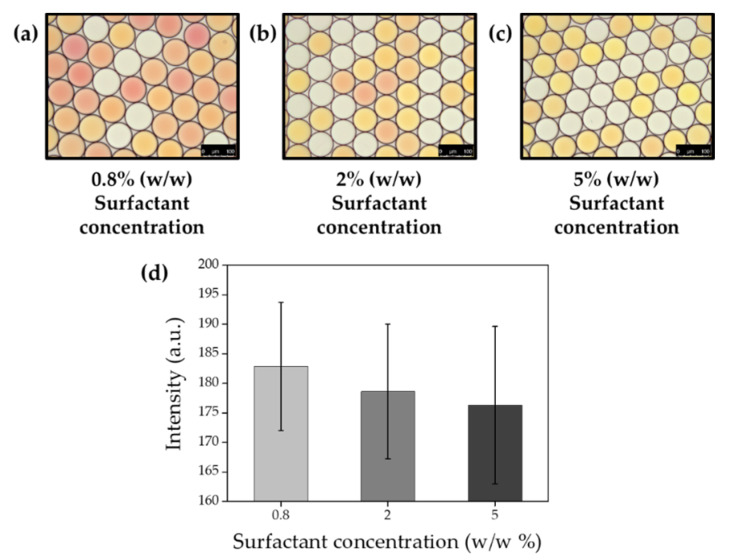
Relationship between surfactant concentration and gas permeability. Microscopic images of droplet mixtures that were generated with HFE-7500 contains 0.8% (**a**), 2% (**b**) or 5% (**c**) surfactant, respectively. (**d**) Mean values of color exchange of indicator droplets for surfactant concentration. (scale bar = 100 μm).

To identify the effect of surfactant concentration, 0.02 M sodium hydroxide and 0.02 M ammonium chloride were co-encapsulated with HFE-7500 that contains different concentrations of surfactant at 0.8, 0.2 and 5%. Collected ammonia gas generating droplets (10 μL) were then mixed with indicator droplets (10 μL, 1 mg/mL phenol red), and mixtures of droplets were immediately transferred into chamber slide for imaging under DMi-8 microscope. Microscopic images were analyzed with the image J software, and the mean values of intensity were fitted with OriginPro software (version 9.0, OriginLab, Northampton, MA, USA) (Figure 14). All procedures were performed at room temperature (23 °C) in this experiment.

### 2.11. Time-Course Measurement of Ammonia Gas Crosstalk through Droplet Barrier

**Figure 15:**
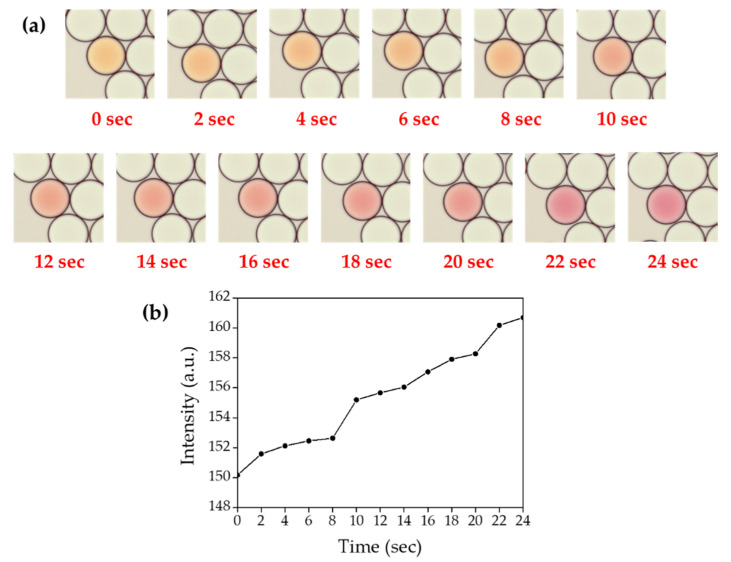
Time-course measurement of ammonia gas crosstalk between droplets. (**a**) Microscopic images of droplet mixture between indicator droplet and ammonia gas generating droplets, the video was recorded for 24 s under DMi-8 microscope. (**b**) Mean values of color change in the indicator droplets.

To measure the time-course transfer of produced ammonia gas across the droplet barrier, 0.02 M sodium hydroxide and 0.02 M ammonium chloride were co-encapsulated with HFE-7500 containing 2% surfactant. Indicator droplets also were also prepared by encapsulating 1 mg/mL phenol red. Ammonia gas generating droplets, oil and indicator droplets were sequentially introduced into the chamber slide. Once the indicator droplet contacted the ammonia gas generating droplets, a video was recorded with LAS X software under a DMi-8 microscope and the color change was identified with image J software (Figure 15). All procedures were performed at room temperature (23 °C) in this experiment.

## 3. Results

### 3.1. Leaking of the Droplets during Determination of Bacteria with pH Indicator

Recently, DMF became a prestigious tool for the monitoring of various pathogens such as bacteria, viruses and fungi at a single-cell scale. In this role, the surfactant is one of the important components because it is needed to contribute to providing stability during droplet generation and reactions such as thermocycling. In this study, the PFPE–PEG–PFPE surfactant was synthesized in two steps (Figure 1), and it was identified prior to droplet generation with bacteria and pH indicator because this surfactant is one of the most commonly used in DMF studies. Synthesized surfactant was analyzed with ATR-FTIR spectroscopy (Figure 2), ^1^H-NMR (Figure 3) and ^19^F-NMR spectroscopy (Figure 4) to identify its chemical structure. In the ATR-FTIR spectrum (Figure 2), CH_2_ stretching, bending bands and C-N stretching bands were respectively indicated at 2876 cm^−1^, 1460 cm^−1^ and 1180 cm^−1,^ which originated from the Jeffamine ED-900. C–F and C-O stretching bands were indicated at 1228 cm^−1^ and 1120 cm^−1^ that originated from Krytox 157-FSH. The C=O stretching frequency in amide bond has been found around 1724 cm^−1^. PFPE acid chloride or PFPE carboxylic acid were not observed when the final product was identified with ATR-FTIR. These results demonstrate that initiator or chemical intermediates were well-removed during synthesis and also cleaning procedures.

In the ^1^H-NMR spectrum (Figure 3), the peak of 5.73 ppm (red circle) is assigned from the amide bond of N-H, and the peak of 3.81 ppm (Blue circle) is assigned from the ether (ROCH_2_R). The peak of 1.2 ppm (green circle) is indicating methylene (CH_2_). As shown in Figure 4, the peaks originated from the PFPE group were assigned as follows: −73 ppm (Pink circle), −80.26 ppm (yellow circle), −80.55 ppm (purple circle), −86.71 ppm (green circle), −125.85 ppm (blue circle), −126.27 ppm (red circle) and −186.96 ppm (orange circle) from the ^19^F-NMR spectrum.

In previous reports, gas permeability was discussed and mentioned, but there was no previous investigation on gas crosstalk between droplets in our understanding. This is why we studied gas permeability through the droplet barrier when droplets were created using HFE-7500 with PFPE–PEG–PFPE surfactant. In Figure 5a, a schematic diagram of the microfluidic device is shown that contains two inlets for the aqueous phase and a single inlet for the oil phase. Droplets were generated through the flow-focusing structure (blue box, Figure 5b), and generated droplets can be incubated within incubation channels (orange box, Figure 5b) prior to the collection through outlet (green–box, Figure 5c). In this study, generated droplets are approximately 80 μm in diameter, which is about 270 pL in volume.

From the beginning of this study, we hypothesized that live bacteria could be determined during cultivation inside droplets with pH indicators because bacterial cells produce ammonia gas as a metabolite, which makes basic pH conditions in cultivation media. In this study, phenol red was co-encapsulated as a pH indicator because it is one of the most commonly used pH indicators for mammalian cell culture, which can turn red in a basic condition. More briefly, single *E. coli* K-12 can be grown within droplets with LB broth, and phenol red can be turned in red due to ammonia gas, which is produced from *E. coli* K-12 during bacterial growth (Figure 6). As shown in Figure 7a,b, there was no color exchange without bacterial growth in droplets. Once single *E. coli* K-12 proliferated within droplets, exponentially grown, *E. coli* K-12 population can be observed under a microscope after overnight incubation (Figure 7g). However, pH indicator (phenol red) was turned in red even droplet does not contain *E. coli* K-12 (Figure 7h). These results demonstrate that pH indicator or bacterial metabolites can penetrate neighber dropletsthrough the droplet barrier. However, it is not clear which components can be transferred between droplets.

### 3.2. Identification of Crosstalk between Droplets

To determine which components across the droplet barrier among pH indicator or bacterial metabolite, an additional experiment was designed, as shown in Figure 8. To identify which metabolite can be transferred into indicator droplets (phenol red), phenol red or *E. coli* K-12 were encapsulated prior to mix (above panel, Figure 8). The result showed that indicator droplets turned red after overnight incubation that suggests bacterial metabolites could cross through the droplet barrier. However, there was no color change from *E. coli* K-12-containing droplets that suggest phenol red cannot penetrate into *E. coli* K-12-containing droplets (Figure 9a,c). *E. coli* K-12 and phenol red were then co-encapsulated within the same droplets to identify the permeability of phenol red through the droplet barrier into empty droplets (below panel, Figure 8). After incubation, phenol red turned red due to bacterial metabolites, which makes basic condition, but there was no phenol red observed from empty droplets (Figure 9b,d). These results also strongly demonstrate that there is no permeability of phenol red between droplets, and only bacterial metabolites can be transferred through the droplet barrier.

Even if ammonia gas production is reported in previous reports [24] and color change of phenol red indicates pH change in basic condition within droplets, it was not clearly identified that ammonia gas is the metabolite crosstalk between droplets. To validate this, the production of ammonia gas during bacterial growth, bacterial metabolite was determined with Nessler’s reagents, which is the conventional indicator of ammonia gas (Figure 10). Phenol red turned red with *E. coli* K-12 culture in LB broth (Figure 10b,e), and Nessler’s reagent also immediately reacted with *E. coli* K-12-containing LB broth (Figure 10c,f). These results suggest that pH change was raised due to the production of ammonia gas during bacterial growth and ammonia gas was the metabolite which was reacted with phenol red.

### 3.3. Validation of Ammonia Gas Crosstalk between Droplets

To identify the permeability of ammonia gas through the droplet barrier, sodium hydroxide and ammonium chloride were co-encapsulated within 80 μm droplets for producing ammonia gas (ammonia gas generating droplets) [24]. As shown in Figure 11, we hypothesized that ammonia gas production could be mimicked by co-encapsulating sodium hydroxide and ammonium chloride. If produced ammonia gas crosstalk into phenol red droplets, the pH indicator will turn red. To validate our hypothesis, droplets were separately generated with 2 M sodium hydroxide (Figure 12b) or 2 M ammonium chloride (Figure 12e). Each droplet was then mixed with phenol red droplets (Figure 12a,d,g), but a color change was not observed (Figure 12c,f). However, phenol red droplets turned red when they were mixed with droplets that co-encapsulated with sodium hydroxide and ammonium chloride to produce ammonia gas within droplets (Figure 12i). These results indicate that ammonia gas was produced from the chemical reaction, and also produced gas was transferred through the droplet barrier.

### 3.4. Effects of Ammonia Gas Concentration and Surfactant Concentration on Crosstalk between Droplets

To identify the effect of gas concentration on permeability between droplets, sodium hydroxide and ammonium chloride were co-encapsulated at a different concentration from 0.002 M to 2 M. Collected droplets were then mixed with indicator droplets and imaged immediately. More bright color changes were observed from indicator droplets due to a higher production of ammonia gas (Figure 13). This result indicates that gas crosstalk was increased in a higher concentration of gas.

In previous reports on DMF, PFPE–PEG–PFPE surfactants have been used at a concentration of 2% on average, which is why we have identified gas crosstalk with a 2% surfactant in this report. However, the effect of surfactant concentration also identified whether it affects gas permeability between droplets. To validate this hypothesis, 0.02 M sodium hydroxide and 0.02 M ammonium chloride were co-encapsulated with HFE-7500 that contains different concentrations of surfactant at 0.8, 2 and 5%. Collected ammonia gas generating droplets were then mixed with indicator droplets, and gas permeability was decreased with a higher concentration of surfactant (Figure 14). These results suggest that the droplet barrier became more densely when surfactant concentration was increased, and it decreased gas permeability.

### 3.5. Time-Course Measurement of Ammonia Gas Crosstalk through Droplet Barrier

**Figure 16:**
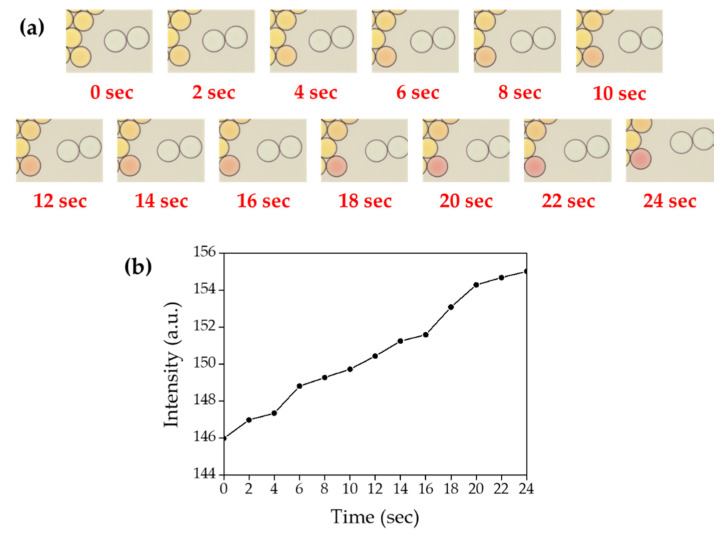
Time-course measurement of ammonia gas crosstalk between droplets. (**a**) Microscopic images of droplet mixture between indicator droplet and ammonia gas generating droplets, the video was recorded for 24 s under a DMi-8 microscope. (**b**) Mean values of color change in the indicator droplets.

To measure the time-course transfer of produced ammonia gas through the droplet barrier, 0.02 M sodium hydroxide and 0.02 M ammonium chloride were co-encapsulated with HFE-7500 containing 2% surfactant prior to mix with indicator droplets. Once the indicator droplet was contacted with ammonia gas generating droplets, it started to turn red in a time-dependent manner (Figure 15, Supplementary Video S1). When sodium hydroxide and ammonium chloride were encapsulated at a concentration of 0.2 M for both chemical compounds, the color change was immediately terminated turned red once the indicator droplet was contacted with ammonia gas generating droplets. We also observed that ammonia gas could be transferred through fluorinated oil even without contact between droplets. As shown in Figure 16 (Supplementary Video S2), phenol red-containing droplets turned red slowly due to gas diffusion through HFE-7500 from two white droplets (that contain 0.2 M sodium hydroxide and 0.2 M ammonium chloride). This result suggested that ammonia gas is soluble in HFE-7500 oil.

## 4. Discussion

The PFPE–PEG–PFPE surfactant is one of the most commonly used surfactants in DMF, and it was mentioned that fluorinated fluids exhibit high gas solubility [23,35]. However, gas crosstalk between droplets has not been validated yet. In this study, we first demonstrated that bacterial metabolic gas penetrates through barriers between droplets, and it contaminates neighboring droplets. Interestingly phenol red dye cannot penetrate the barrier between droplets due to its relatively higher molar mass (354.38 g/mol), but ammonia gas can across the droplet barrier because of its lower molar mass (17.031 g/mol) in our hypothesis.

It is already known that the weak interactions of fluorinated compounds in relatively high compressible fluids result in high gas solubility, so the interfacial surfactant molecules may be the main barriers that affect gas crosstalk [22]. To validate this hypothesis, the relation of surfactant concentration and gas transport was identified. To provide a higher surface density, surfactant concentration was increased up to 5%, and it decreased gas permeability between droplets and this result indicates surfactant density is the potential reason for the gas crosstalk between droplets.

We also confirmed that ammonia gas was produced during cultivation within droplets, and these results suggest that live bacteria can be grown and monitored using DMF by analyzing the production of ammonia gas. Since bacterial contamination is one of the most serious issues in drinking water, bacterial growth can be monitored by the identification of ammonia gas production using DMF without complex and expensive approaches such as real-time PCR. Even droplets were analyzed with an expensive and large microscope in our study; the microscope can be replaced with inexpensive and portable imaging systems, including smartphones and portable CCD cameras.

Basically, leaking of the droplets is the potential issue on analytical approaches, including basic biological studies and diagnosis because some of the interested biomarkers can be penetrated into neighboring droplets if it is small enough. Since contamination can be a potential reason for false-positive droplets, this issue must be eliminated in case of various diagnosis purposes. Even large-sized, highly hydrophobic and highly hydrophilic components have less chance for crosstalk through the droplet barrier; there is still potential that tiny components can be a useful biomarker for diseases. It turns that a dense droplet barrier must be constructed, which means stronger surfactants must be required for DMF in the future. However, leaking also can be a useful tool that can provide internal components to neighboring droplets. For example, bacteria droplets can be a tiny microcapsule that can supply ammonia gas into the microsized reactor that requires ammonia gas for the reactions such as chemical synthesis, analytical procedures and catalytic reactions.

## Supplementary Materials

The following are available online at https://www.mdpi.com/2079-6374/10/11/172/s1, Video S1: Gas crosstalk between droplets when droplets are contected. Video S2: Gas crosstalk through fluorinated oil without contact between droplets.
